# Targeting Autophagy with Natural Compounds in Cancer: A Renewed Perspective from Molecular Mechanisms to Targeted Therapy

**DOI:** 10.3389/fphar.2021.748149

**Published:** 2021-08-26

**Authors:** Qiang Xie, Yi Chen, Huidan Tan, Bo Liu, Ling-Li Zheng, Yandong Mu

**Affiliations:** ^1^ Department of Stomatology, Sichuan Provincial People’s Hospital, University of Electronic Science and Technology of China, Chengdu, China; ^2^ Department of Stomatology, Zigong First People’s Hospital, Zigong, China; ^3^ Department of Gastrointestinal Surgery, State Key Laboratory of Biotherapy and Cancer Center, West China Hospital, Sichuan University, Chengdu, China; ^4^ Department of Pharmacy, The First Affiliated Hospital of Chengdu Medical College, Chengdu, China

**Keywords:** autophagy, natural compound, pathway, cancer therapy, small-molecule drug

## Abstract

Natural products are well-characterized to have pharmacological or biological activities that can be of therapeutic benefits for cancer therapy, which also provide an important source of inspiration for discovery of potential novel small-molecule drugs. In the past three decades, accumulating evidence has revealed that natural products can modulate a series of key autophagic signaling pathways and display therapeutic effects in different types of human cancers. In this review, we focus on summarizing some representative natural active compounds, mainly including curcumin, resveratrol, paclitaxel, Bufalin, and Ursolic acid that may ultimately trigger cancer cell death through the regulation of some key autophagic signaling pathways, such as RAS-RAF-MEK-ERK, PI3K-AKT-mTOR, AMPK, ULK1, Beclin-1, Atg5 and p53. Taken together, these inspiring findings would shed light on exploiting more natural compounds as candidate small-molecule drugs, by targeting the crucial pathways of autophagy for the future cancer therapy.

## Introduction

The term of autophagy was first reported in 1963 (Yang and Klionsky, 2010), with gradually understanding that autophagy plays a role in the regulation of human life activities. Autophagy, through its conservative mechanism, decomposes macromolecules and provides a lot of nutrients, so it plays an irreplaceable role in regulating metabolism and cell growth (Yan et al., 2019). Importantly, autophagy has dual effect on cancers, which may protect cancer cells from extreme nutrient conditions, while it also causes destruction of energy homeostasis and kill cancer cells (Chen et al., 2021). In physiological state, autophagy maintains cell homeostasis by degrading and removing damaged or dead organelles, misfolded proteins and other substances, while abnormal autophagy will break the original balance of cells, and even play a key role in the occurrence and development of cancer (Byun et al., 2017). Herein, a deep understanding of the biological relationship between autophagy and cancer is critical to explore potential targets for cancer treatment and provide new clues for anti-cancer drug development.

As we all know, natural compounds (refer to the small and large molecules extracted and separated from natural products) come from a variety of sources, including plants, animals, and even microbes. For example, curcumin, a diketone compound extracted from the rhizomes of some plants in the family Zingiberaceae and Araceae, and oridonin is a bioactive diterpenoid compound isolated from *Rabdosia* (Iabtea). In addition, there is a secretion from the animal toad - Bufalin. Accumulating evidences have shown that natural compounds play an important role in the treatment of cancer, including inhibiting cancer cell proliferation and inducing apoptosis, as well as inhibiting cancer cell metastasis and angiogenesis (Liu et al., 2019a; Sun et al., 2019; Liu et al., 2020; Kang et al., 2021). More interestingly, in the treatment of tumors, multiple natural compounds are reported to exert therapeutic effect by targeting autophagy (Towers et al., 2020). For example, Resveratrol has been shown to induce protective autophagy and exert anticancer activity in non-small cell lung cancer by inhibiting Akt/mTOR and activating the P38-MAPK pathway (Wang et al., 2018b). Additionally, the antitumor bioactivity of curcumin is achieved by inhibiting PI3K/Akt/mTOR pathway and inducing apoptosis and autophagy of human lung cancer A549 cells (Liu F. et al., 2018).

In different types of tumors, natural compounds induce or inhibit autophagy and thus suppress tumor growth through a variety of different mechanisms of action. Notably, the exact mode of autophagy modulated by natural compounds derived from Mother Nature is highly complex, it needs to be further studied and explored deeply. Therefore, we write this review to clarify the complex biological relationship between natural compounds, autophagy, and cancer, in order to provide new ideas for the development of anticancer drugs.

## Autophagic Process

Autophagy is a continuous process of dynamic development, mainly including the initial stage, extension stage and mature degradation stage of autophagosome, a bilayer membrane structure which fuses into lysosome, and further form autophagolysosomes to degrade the material encapsulated in it and release it for reuse. The autophagic process is regulated by many factors, and more than 40 autophagy related genes (ATG) and corresponding proteins have been found to participate in autophagy regulation (Chou et al., 2021; Ferreira et al., 2021).

Different from the lower autophagy level under normal physiological conditions, when cells undergo starvations, autophagy may produce intermediate nutrients for cells survival, which was exploit by cancer cells to establish chemoresistance and adapt nutrient depletion. On the contrary, some mutations of oncogenes and epigenic modifications occurring in cancer may negatively regulate autophagic level and suppress carcinogenesis, representing a tumor-suppressive role of autophagy (Morselli et al., 2009; Arroyo et al., 2014). Therefore, autophagy has the dual effects in the promotion and inhibition of cancer with cytotoxic and cytoprotective effects on tumor cells.

### The Janus Role of Autophagy in Cancer

Autophagy could promote tumor progression under low-oxygen and low-nutrient conditions. Meanwhile, autophagy may exert a tumor suppressor effect through a variety of mechanisms, so the role of autophagy in cancer development appears to be paradoxical and complex. Depending on different environments, such as tumor type, stage and stress type, autophagy regulates pro-survival or pro-death signaling pathways in cancers (Singh et al., 2018).

At the molecular level, the occurrence and development of tumors involve numerous signaling pathways, and the signaling pathways of autophagy are still emerging, mainly involving protein 53 (p53), B cell lymphoma/leukemia-2 (Bcl-2), Akt, Bax-interacting factor-1 (Bif-1), Ras, mammalian rapamycin target (mTOR) and phosphoinositide 3-kinase (PI3K) I (Khandia et al., 2019). Moreover, these signaling pathways are often associated with each other, and can be integrated into the tumor regulatory network of autophagy related proteins, which ultimately affect the fate of tumor cells (Morselli et al., 2009).

### Tumor Suppressive Role of Autophagy

At present, it is generally believed that autophagy that appears in the early stage of tumorigenesis has an inhibitory effect. Spontaneous tumors are found in Beclin-1 ± mouse, indicating that defective autophagy may promote tumorigenesis. In addition, research on Beclin-1, which is a common haploin-sufficient tumor suppressor missing in ovarian, breast and prostate cancers, establishes the first direct functional connection between cancer and autophagy and supports the theory that autophagy can inhibit the formation of initial tumors. In addition, deletions of other several Atg genes besides Beclin-1 have also been found to promote tumorigenesis. For example, systematic deletion of liver-specific Atg7 and Atg5 in mice will lead to an enhancement in the formation rate of liver tumors. These researches on the effect of defective autophagy on cancers using Atgs gene deletion mice support that autophagy suppresses tumor formation in the early stage. In addition, allelic deletion of Beclin-1–interacting proteins, such as Bif-1 and UVRAG also occurs in various cancers, and changes in the expression of them can increase the incidence of spontaneous cancer by altering the autophagy pathway. Beclin-1 synergistically activates lipid kinase vacuolar protein sorting 34 (Vps34) with activating molecule in Beclin-1-regulated autophagy (Ambra-1), Bif-1 and UVRAG to induce autophagy (Brech et al., 2009). However, in the lysosomal degradation pathway of autophagy, it can inhibit tumor function.

Forkhead box O (FOXO) is a key autophagy regulator that regulates cell proliferation and apoptosis by activating autophagy activity. FOXO1 regulated cell death may be related to tumor inhibitory activity (Zhao et al., 2010; Ling et al., 2011). The expression of tumor suppressor FOXO3a is usually down regulated in cancer (Liu Y. et al., 2018). Activated FOXO3a may induce autophagy *via* enhancing the transcription of autophagic regulators including BNIP3 and LC3, while mTORC2 blocks the activation of FOXO3a by activating Akt (Ni et al., 2013). Therefore, FOXO3a is a key molecule connecting autophagy and cancer, and its expression may be regulated by autophagy-related signaling pathways, and therefore it is hopeful that it will become a therapeutic target for cancer.

Sirtuin-1 (SIRT1), as a NAD^+^-dependent deacetylase, mainly regulates the deacetylation of FoxO (Wang and Tong, 2009; Kim et al., 2012; Hu et al., 2017). Under stress conditions, the complex of FOXO1 and sirtuin-2 (SIRT2) was dissociated and then acetylated and acetylated FOXO1 is balanced by histone acetylase and histone deacetylase (HDAC). FOXO1-Atg7 complex affects the autophagy process and eventually leads to cell death.

### Oncogenic Role of Autophagy

When exposed to the two induction conditions of either hypoxia or nutrient deficiency, damaged organelles and unnecessary proteins can be decomposed by autophagy, and thereby promoting the viability of cancer cells, which indicates that autophagy can promote tumor growth and progression. In addition, in some cases, autophagy can be activated through some carcinogenic pathways. For example, oncogene pathways such as mTOR, Akt, PI3KCI, Bcl-X_L_, Bcl-2, BCR-ABL, and Ras play an irreplaceable role in determining the survival of cancer cells. In pancreatic colorectal tumors with high RAS (rat sarcoma) gene mutation frequency, the level of autophagy is observed to be correlatively high, where the increase of autophagy helps to maintain the proliferation of cancer cells. The dependence of tumor growth on autophagy similar to that of RAS-driven cancers has also been observed in lung cancer, which was due to the substitution drive of valine to glutamate at BRAF position 600 (BRAFV600E). Of note, the activation of some oncogenes such as kirsten rat sarcoma viral oncogene (KRAS) or the deletion of tumor suppressor genes such as p53 have been used to form spontaneous tumors to establish genetically engineered mouse tumor models (GEMMs).

Sustained Ras-Raf-MAPK may be necessary to maintain tumor survival through autophagy. Importantly, mTOR is the main negative regulator of tumor cell autophagy, which can be activated by Ras-Raf-1-MEK1/2-ERK1/2 and PI3KCI-Akt signaling pathways, while inhibited by kinase B1 (LKB1)-AMP-activated protein kinase (AMPK) pathway (Wang et al., 2011). mTOR pathway regulates autophagy through two mechanisms. MTORC1 acts on EIF-4E Binding Proteins (EIF4EBPs) and S6K1 through signal transduction pathway, which may start the transcription and translation of related genes and regulate autophagy (Wang and Zhang, 2019). mTOR kinase may also act directly on Atg and regulate autophagy (Petherick et al., 2015). As a negative regulator of translation that can be phosphorylated by mTORC1, 4E-BP1 is inactivated and separated from eIF-4E after phosphorylation, so as to activate eIF-4E. EIF-4E can affect cancer progression by regulating the translation of a variety of proteins including RAS and cyclin D-MYC, which are closely related to the proliferation, cell cycle regulation and growth of cancer cells. (Huang et al., 2019; Joseph et al., 2019). In addition, BCR-ABL may function as a key factor to stimulate mTOR transcription in chronic myeloid leukemia (CML) through PI3KCI-Akt-FOXO signaling (Kang et al., 2011).

## Natural Compounds as Autophagic Modulators in Cancer

Based on the duality of autophagy in tumor cells, the current development and research of autophagy regulatory drugs mainly includes autophagy inducers and autophagy inhibitors. This section focuses on several representative natural compounds including Curcumin, Paclitaxel, Resveratrol, Bufalin and Ursolic acid, and summarize the autophagy-regulating mechanisms of other compounds (Supplementary Table 1).

### Curcumin

**FIGURE 1:**
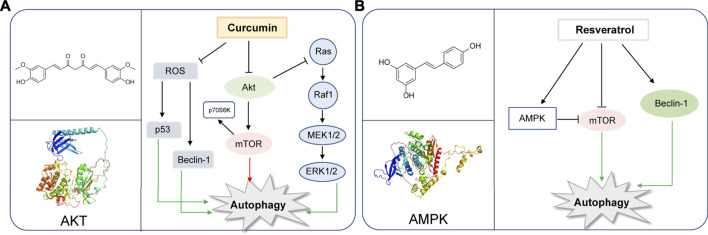
Representative natural compounds targeting autophagic pathways in cancer **(A)** Curcumin **(B)** Resveratrol.

Curcumin is a chemical component extracted from the rhizomes of some plants of Zingiberaceae and Araceae. Many preclinical studies *in vitro* and *in vivo* have proved that curcumin is safe and effectual in a variety of cancers. In recent years, curcumin was found to regulate autophagy, active apoptosis and inhibit the proliferation of tumor cells. (Li et al., 2020). The autophagy-related molecules such as Beclin-1, mTOR, ERK1/2, P53, play important roles in the initiation of autophagy (White, 2016; Xu et al., 2018; Shi et al., 2019). In human ovarian cells, lung cancer cells and human melanoma cells, curcumin inhibits the Akt/mTOR/p70S6K pathway to induce protective autophagy (Zhao et al., 2016; Liu et al., 2019b; Zhang et al., 2019). Curcumin is also found to activate the ERK1/2 pathway and inhibit the Akt/mTOR/p70S6K pathway and activate the ERK1/2 pathway to induce autophagy in malignant glioma cells (Aoki et al., 2007). Moreover, curcumin stimulates the p38 MAPK and phosphorylation of ERK1/2 in malignant mesothelioma cells (Masuelli et al., 2017). In SiHa cells and HCT116 cells, curcumin is found to upregulate p53 and Beclin-1 and induce reactive oxygen species (ROS) in activation of autophagy (Lee et al., 2011; Wang T. et al., 2020). Besides that, curcumin exerts its antitumor action inhibiting Bcl-2 and elevating the expression of Bax, p53, pro-caspases 3, 8, and 9 (Kuttan et al., 2007). YAP and P62 are observed to be reduced and LC3-Ⅱis enhanced with the treatment of curcumin in HCT116 and SW620. The results reveal that curcumin promotes autophagy and inhibits the proliferation of colon cancer cells (Zhu et al., 2018). Recently, studies have suggested that curcumin can modulate the expression of miRNAs, which induce apoptosis and suppress tumorigenesis and metastasis (Norouzi et al., 2018). Also, in human prostate cancer, curcumin could sensitize them to radiation by autophagy inhibition through miR-143 mediated pathway (Liu et al., 2017). Additionally, curcumin downregulates Bcl-xL by interfering with the PI3K/Akt and NF-κB signal pathways, as well as inducing the mitochondrial dysfunction together with caspase-3 activation (Liu et al., 2007) (Figure 1A).

### Resveratrol

Resveratrol, a constituent of red wine, is reported to exert therapeutic potential on various tumors, whose mechanism is closely associated with the regulation of autophagy (Fremont, 2000; Baur and Sinclair, 2006; Deng et al., 2019). Resveratrol was reported to induce autophagy by direct inhibition of mTOR-ULK1 pathway in an ATP-competitive way, and can kill MCF7 cells which are mTOR inhibitor sensitive, displaying anti-cancer potential (Park et al., 2016). Resveratrol may induce autophagic cell death by activating c-Jun N-terminal kinase (JNK) pathway, as well as inducing AMPK pathway and ultimately leading to inhibition of mTOR pathway (Puissant et al., 2010).Resveratrol was reported to trigger autophagy through suppressing Akt/mTOR and inducing p38-MAPK pathways in human non-small-cell lung cancer cells A549 and H1299, thereby hindering cancer cell proliferation (Wang et al., 2018b). Additionally, resveratrol evokes autophagic cell death in a stromal interaction molecule 1 (SIMI)-dependent way, while involving the downregulation of mTOR pathway in human prostate cancer cells PC3 and DU145 (Selvaraj et al., 2016). When combined with quercetin (QCT), resveratrol reduced the QCT-induced autophagy through AMPK phosphorylation and heme oxygenase 1 (HO-1) downregulation, sensitizing the therapeutic effect of QCT (Tomas-Hernandez et al., 2018). Notably, resveratrol was recently used as supplement drug for chemotherapeutic anti-cancer drugs such as temozolomide (TMZ) to enhance the therapeutic effect (Lin et al., 2012). In gliomas, resveratrol suppressed cytoprotective autophagy which is triggered by TMZ treatment through inhibiting the ROS/ERK pathway, thus enhancing the efficacy of TMZ. Moreover, combined treatment of resveratrol and TMZ may significantly reduce the tumor volume through suppressing the ROS/ERK pathway than either TMZ or resveratrol alone, suggesting the synergistic effect of these two drugs (Lin et al., 2012). In cisplatin-resistant human oral cancer CAR cells, resveratrol activates autophagy *via* regulating AMPK and Akt/mTOR pathways, by phosphorylating AMPKα on Thr172 and AMPKα and dephosphorylating Akt on Ser473 and mTOR on Ser2448, and ultimately inhibits CAR cell viability (Chang et al., 2017) (Figure 1B).

### Paclitaxel

**FIGURE 2:**
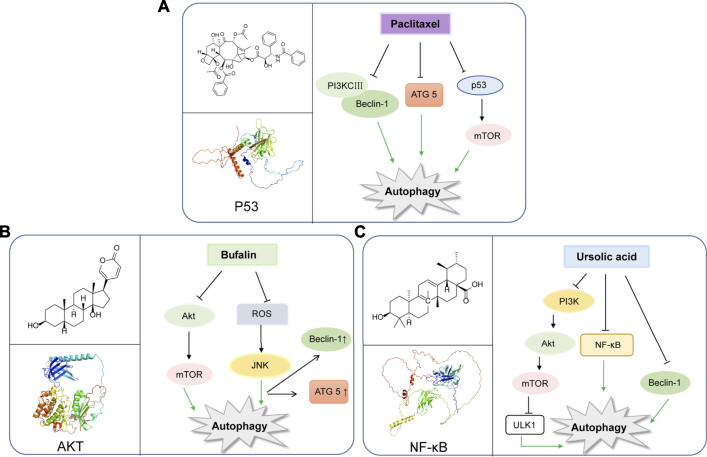
Representative natural compounds targeting autophagic pathways in cancer **(A)** Paclitaxel **(B)** Bufalin **(C)** Ursolic acid.

Paclitaxel is known as a plant alkaloid extracted from the dry root, branches, leaves, and bark of the Taxus (Wessely et al., 2006). Nowadays, paclitaxel has been a first-line drug for plenty of cancers, and greatly help improve patient survival in lung, ovarian and breast cancer (Yu et al., 2015). In a study, paclitaxel could be applied for metastatic or locally advanced breast cancer by inducing autophagy in lymphatic endothelial cell (LEC). This process was independent from lymph node lymphangiogenesis because paclitaxel can only strongly inhibit lymphangiogenesis in combination with autophagy inhibitors such as chloroquine (Zamora et al., 2019). Moreover, paclitaxel treatment can induce autophagy by increasing expression levels of Atg5 and Beclin-1, which are essential to autophagy initiation in the A549 cells (Xi et al., 2011). In breast cancer, paclitaxel could induce early autophagy and is associated with apoptosis (Notte et al., 2013). Interestingly, paclitaxel exerts the same function in cervical cancer (Zou et al., 2018). Hitherto, paclitaxel has been shown to induce autophagy in BGC823 gastric cancer cell line, thus playing an anticancer role in inhibiting cell proliferation (Yu et al., 2017). In addition, multidrug resistance is a great challenge for paclitaxel to exert effect on cancers (Wang et al., 2018a). While, it was found that esomeprazole could overcome drug resistance of paclitaxel and enhance its anticancer effects by inducing autophagy in A549/Taxol cells (Bai et al., 2021). In a randomized phase II preoperative study, combination of autophagy inhibitor hydroxychloroquine and gemcitabine and nab-paclitaxel could exert autophagy inhibition in pancreatic cancer patients (Zeh et al., 2020) (Figure 2A).

### Bufalin

Bufalin was found to be a cardiac steroid derived from posterior auricular glands and the skin of *Bufo gargarizans*. As an active ingredient in traditional Chinese medicine, Bufalin has been shown to be biologically active against various cancers (Zhang et al., 1992; Lee et al., 2017; Wu et al., 2017; Yang et al., 2018). Bufalin has been reported to inhibit glycolysis-induced ovarian cancer cell growth and proliferation *via* the suppression of Integrin β2/FAK signaling pathway (Li H. et al., 2018). In addition, bufalin could induce cell death through reactive oxygen species mediated RIP1/RIP3/PARP-1 pathway, thus exerting pharmacological activity to inhibit the development of human breast cancer (Li Y. et al., 2018). Notably, autophagy plays an important role in Bufalin’s anti-tumor biological function, and it induces autophagy-mediated cell death through ROS production and JNK activation in colon cancer (Xie et al., 2011). In recent years, Bufalin has also been shown to induce autophagy in gastric cancer MGC803 cells through Akt/mTOR/P70S6K and ERK signaling pathways. It is worth mentioning that Cbl-b mediated inhibition of mTOR and activation of ERK1/2 may play key roles. (Qi et al., 2019). Bufalin and JNK pathway induced autophagy, which was closely related to down-regulation of Bid and BCL-2, and up-regulation of MAPK, TNF, ATG8 Beclin-1 (Hsu et al., 2013). Moreover, Bufalin also activated autophagy in HepG2 cells, enhancing LC3-I to LC3-II transformation and Beclin-1 expression, and decreasing mTOR signal activation and p62 expression. Intriguingly, inhibition of autophagy by selective inhibitor 3-MA decreased the rate of apoptosis of HepG2 cells treated with Bufalin, which indicated that Bufalin-induced autophagy could promote apoptosis (Miao et al., 2013). Interestingly, Bufalin not only induces apoptosis through autophagy, it also plays a crucial role in anti-metastasis and anti-invasion. Bufalin has been found to inhibit the metastasis and invasion of hepatoma cells *via* PI3K/Akt/mTOR signaling pathway (Sheng et al., 2021) (Figure 2B).

### Ursolic Acid

Ursolic acid (UA) is well-known as a triterpenoid compound enriched in various plants, which has been widely reported to exhibit its antitumor activity by modulating autophagy in different types of cancers. And, it can inhibit breast cancer cell proliferation through the PI3K-Akt and NF-κB pathways, leading to autophagy and apoptosis (Luo et al., 2017). In addition, UA can also promote cytotoxic autophagy and apoptosis, associated with AMPK and ERK1/2 signaling pathways by targeting glycolysis in different types of breast cancer cells (Lewinska et al., 2017). Additionally, UA also shows the inhibition of cell migration and induces JNK-dependent lysosomal associated cancer cell death, which is similar to autophagy in glioblastoma multiforme cells (Conway et al., 2021). Moreover, UA modulates autophagy and apoptosis in oral cancer cells through the Akt-mTOR and NF-κB pathways (Lin et al., 2019). Interestingly, UA induces mitophagy *via* the Akt-mTOR signaling pathway and is dependent on PINK1 in A549 human lung cancer cells. And, UA induces a Reactive Oxygen Species production by regulating p62 and Nrf2. (Castrejon-Jimenez et al., 2019). Similarly, UA is also reported to inhibit metastasis of esophageal cancer cells by ROS-mediated autophagy (Lee et al., 2020). Additionally, UA induces apoptosis and inhibits autophagy progression, leading to PC-12 cell death (Jung et al., 2018). Also, UA can inhibit pigmentation by activating B16F1 cell melanosomal autophagy (Park et al., 2020). More importantly, autophagy (cytoprotective autophagy) inhibition has been recently reported to enhance the anti-tumor effects of UA on lung cancer cells by the mTOR (Wang M. et al., 2020). More importantly, in gemcitabine-resistant human pancreatic cancer cells, UA has been shown to induce apoptosis, autophagy, and chemosensitivity (Lin et al., 2020). Combined use of Zoledronic acid can augment UA-induced apoptosis *via* enhancing oxidative stress and autophagy in osteosarcoma cells, indicating a potential combination therapeutic strategy for UA (Wu et al., 2016). Taken together, the above-mentioned findings may elucidate the potential underlying molecular mechanisms of UA and provide a novel therapeutic strategy to improve cancer treatment. (Figure 2C).

## Other Natural Compounds Targeting Autophagy in Cancer

In addition to the above five compounds, other natural compounds have also been reported in recent years to regulate autophagy and induce cancer cell death. Genistein could promote cancer cell death through autophagic activation by inhibiting Akt in the treatment of many tumor types (Gossner et al., 2007). Additionally, fixetine can regulate autophagy by acting as an inhibitor of PI3K/Akt/mTOR pathway in human NSCLC cells and prostate cancer (Sun et al., 2018). Angelica sinensis is psoralen, derived from Angelica sinensis polymorph, which has been proved to regulate autophagy and apoptosis (Rahman et al., 2016; Wang et al., 2019) Angelicin increases autophagic proteins including Atg3, 7 and Atg12-5 through the phosphorylation of mTOR (Wang et al., 2019; Uddin et al., 2020). In addition, Camptothecin, vincristine, podophyllotoxin, Betulinic acid and other Changchun herbs can trigger autophagy in multiple myeloma cells, breast cancer cells, colon cancer cells and other different types of tumors (Deng et al., 2019; Chen et al., 2021; Pang et al., 2021).

## Therapeutic Potential of Natural Compounds in Cancer

As we have known, autophagy plays a dual role in cancer. Natural compound could promote or inhibit autophagy to treat cancer cell. In the past decades, numerous natural compounds have been studied their function on cancer treatment by targeting autophagy. Some key regulators of autophagy, such as mTOR, Beclin-1, p53, Akt, ERK, NF-κB and reactive oxygen species (ROS) have been the target for natural compounds to modulate cancer development and exhibit therapeutic effects against various cancers. For example, regulation of PI3K/Akt/mTOR signaling is a classic pathway involved in autophagy regulation, thus G-protein-coupled receptor antagonists, PI3K inhibitors, Akt inhibitors and mTOR inhibitors can inhibit this signaling pathway and induce autophagy in cancer therapy. In addition, it has been reported that Beclin-1 plays a key role as an essential gene for autophagic vesicle formation, therefore these compounds also regulating Beclin-1 in autophagic cell death. Meanwhile, Bcl-2 and Bcl-xL may inhibit autophagy activation by binding to Beclin-1. Thus, natural compounds, such as gossypol, potently bind to Bcl-2 and Bcl-xL and release Beclin-1 to preferentially inducing autophagy. Of note, a series of natural compounds have also shown strong anti-tumor efficacy by directly targeting ROS production through multiple signaling pathways involved in the interaction of ROS and autophagy. All above, increasing autophagy pathways of natural compounds have been discovered and show anti-tumor effect by regulating these signaling. Therefore, natural compounds have a great clinical prospect in cancer therapy by regulation of autophagy. With the discovery of more new natural products, it will provide opportunities for the discovery of new anticancer drugs.

## Concluding Remarks

Hitherto, autophagy has been widely considered to have a dual role in tumorigenesis, in which defective autophagy may facilitate cancer development whereas cytoprotective autophagy helps cancer cell escape stress including starvation and hypoxia and ultimately promote the progression of cancer. Growing numbers of natural compounds have been reported to modulate multiple signal pathways associated with autophagy, such as Ras-Raf-MEK-ERK, PI3K-Akt-mTOR and AMPK, thus exerting therapeutic effect on different cancers. Notably, natural compounds are multiple-targets and less toxic, and represent promising candidates for treating cancer. In this review, we focus on five widely-investigated compounds in regulating autophagy, namely curcumin, resveratrol, paclitaxel, Bufalin, and Ursolic acid, and summarize their corresponding molecular mechanism of regulating autophagy. However, plenty of studies on these natural compounds only investigate their anti-cancer effects *in vitro*, lacking experimental data of *in vivo* studies. However, with the further research, animal model validation and other methods will effectively solve these problems, and greatly promote the study of natural compounds pharmacological mechanism.

Interestingly, most of these natural compounds not only regulate autophagy, but also have effects on apoptosis, so they can be good candidates to target the Regulated Cell Death (RCD) network, which is a hot and promising researching field. Moreover, due to their modulative role of autophagy, they exhibit good property to supplement classical chemotherapy drugs and help to overcome drug resistance. Also, combination of natural compounds is benefit to reduce the effective dose and enhance efficacy, representing a future research direction. In summary, our review on natural compounds targeting autophagy in cancer therapy may shed new light on exploiting more natural compounds and provide clues for developments of anti-cancer drugs.

## Abbreviations

Ambra-1, Activating molecule in Beclin-1-regulated autophagy; AMPK, AMP-activated protein kinase; Atg, Autophagy related genes; Bcl-2, B cell lymphoma/leukemia-2; Bif-1, Bax-interacting factor-1; BRAFV600E, BRAF position 600; CML, Chronic myeloid leukemia; EIF4EBPs, EIF-4E binding proteins; FoxO, Forkhead box O; GEMMs, Genetically engineered mouse tumor models; HDAC, Histone deacetylase; HO-1, Heme oxygenase 1; JNK, c-Jun N-terminal kinase; KRAS, Kirsten rat sarcoma viral oncogene; LEC, Lymphatic endothelial cell; mTOR, mammalian target of rapamycin; p53, protein 53; PI3K, Phosphoinositide 3-kinase; QCT, Quercetin; ROS, Reactive oxygen species; SIMI, Stromal interaction molecule 1; SIRT1, Sirtuin-1; SIRT2, Sirtuin-2; TMZ, Temozolomide; UA, Ursolic acid; VPS34, Vacuolar protein sorting 34.
