# Supplementary material for: Targeting Autophagy with Natural Compounds in Cancer: A Renewed Perspective from Molecular Mechanisms to Targeted Therapy
Source: Front Pharmacol. 2021 Aug 26;12:748149. doi: 10.3389/fphar.2021.748149 (PMC8427500; doi:10.3389/fphar.2021.748149)
Supplement: Supplementary file 1 [file Table1.DOCX]

**Table.S1 Targeting autophagy with some representative natural compounds in cancer**

| **Compound** | **Structure** | **Source** | **Pathways** | **Tumor type** | **Ref.** |
| --- | --- | --- | --- | --- | --- |
| Curcumin |  | The rhizomes of some plants of Zingiberaceae and Araceae. | Akt/mTOR/p70S6K  ERK1/2  PI3K/Akt  NF-κB | Lung cancer, melanoma, malignant glioma, malignant mesothelioma, colon cancer, prostate cancer. | (Li et al., 2020) |
| Paclitaxel |  | The bark of the Pacific yew tree-Taxus brevifolia. | Atg5/Beclin-1 | Breast cancer, cervical cancer, ovarian cancer. | (Wessely et al., 2006) |
| Resveratrol |  | Red wine. | AMPK/mTOR/ULK1  JNK  Akt/mTOR  p38/MAPK  ROS/ERK | Non-small-cell lung cancer, prostate cancer, glioma, oral cancer. | (Baur and Sinclair, 2006; Deng et al., 2019; Fremont, 2000) |
| Bufalin |  | The skin and posterior auricular glands of bufo gargarizans. | Integrin β2/FAK  RIP1/RIP3/PARP-1  AKT/mTOR/P70S6K  ERK  JNK  PI3K/AKT/mTOR | Ovarian cancer, breast cancer, colon cancer, hepatoma. | (Lee et al., 2017; Wu et al., 2017; Yang et al., 2018; Zhang et al., 1992) |
| Ursolic acid |  | Plants. | PI3K/AKT/mTOR  NF-κB  AMPK  ERK1/2 | Breast cancer,  oral cancer, lung cancer, esophageal cancer, pancreatic cancer, osteosarcoma. | (Luo et al., 2017) |
| Genistein |  | Plants | Inhibiting Akt activation | prostate cancer, breast cancer, colon cancer, gastric cancer, lung cancer, pancreatic adenocarcinoma and lymphoma | （Gossner et al., 2007） |
| Fixetine |  | Plants | Inhibiting PI3K / Akt / mTOR pathway expression and regulating autophagy | prostate cancer/breast cancer/lung cancer | (Sun et al., 2018) |
| Angelica sinensis | \ | Angelica sinensis polymorph | Inducing apoptosis and autophagy by increases autophagy related proteins Atg3, Atg7 and Atg12-5 | cancers | (Uddin et al., 2020; Wang et al., 2019). |
| Camptothecin |  | Plants | PI3K/AKT/mTOR | myeloma cells, breast cancer cells, colon cancer cells | (Chen et al., 2021). |
| Vincristine |  | Plants | PTEN/AKT/mTORC1 pathway | myeloma cells, breast cancer cells, colon cancer cells | (Deng et al., 2019). |
| Podophyllotoxin |  | Plants | Promoting cell death via cell cycle arrest, ER stress and autophagy | myeloma cells, breast cancer cells, colon cancer cells | (Pang et al., 2021). |
| Betulinic acid |  | Plants | PI3K/AKT/mTOR  NF-κB/ERK/p53 | myeloma cells, breast cancer cells, colon cancer cells | (Pang et al., 2021). |
